# Supplementary material for: Targeting Glutaminolysis Shows Efficacy in Both Prednisolone-Sensitive and in Metabolically Rewired Prednisolone-Resistant B-Cell Childhood Acute Lymphoblastic Leukaemia Cells
Source: Int J Mol Sci. 2023 Feb 8;24(4):3378. doi: 10.3390/ijms24043378 (PMC9965631; doi:10.3390/ijms24043378)
Supplement: Supplementary file 1 [file ijms-24-03378-s001.zip › Revised_Supplementary File 1 10.02.2023.pdf]

# *Supplementary File*

Figure S1

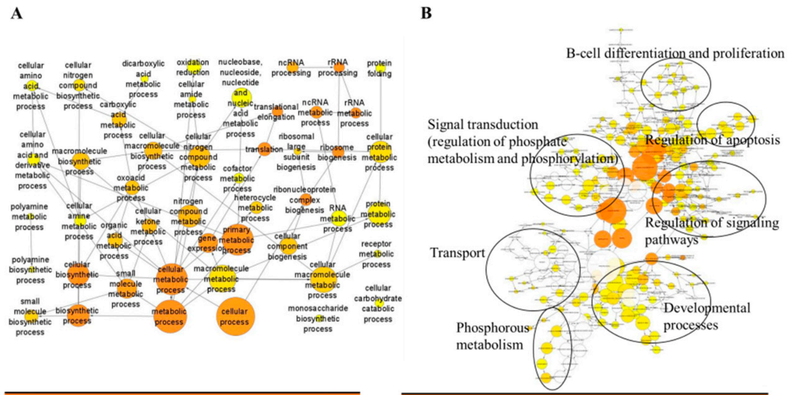


| Enrichment term | p-value |
|---|---|
| cellular metabolic process | $3.09\times10^{-10}$ |
| cellular biosynthetic process | $3.09\times10^{-10}$ |
| ribosome biogenesis | $3.09\times10^{-10}$ |
| biosynthetic process | $1.58\times10^{-9}$ |
| translation | $3.05\times10^{-9}$ |
| metabolic process | $3.05\times10^{-9}$ |
| rRNA processing | $3.45\times10^{-8}$ |
| translational elongation | $3.77\times10^{-8}$ |
| ribonucleoprotein complex biogenesis | $3.88\times10^{-8}$ |
| rRNA metabolic process | $5.79\times10^{-8}$ |

| Enrichment term | p-value |
|---|---|
| signaling | $1.338\times10^{-15}$ |
| signal transduction | $3.449\times10^{-14}$ |
| signaling process | $3.449\times10^{-14}$ |
| signal transmission | $3.449\times10^{-14}$ |
| regulation of signal transduction | $1.748\times10^{-10}$ |
| regulation of signaling process | $2.305\times10^{-10}$ |
| regulation of signaling pathway | $2.305\times10^{-10}$ |
| regulation of cell communication | $2.614\times10^{-10}$ |
| biological regulation | $7.642\times10^{-10}$ |
| signaling pathway | $7.642\times10^{-10}$ |

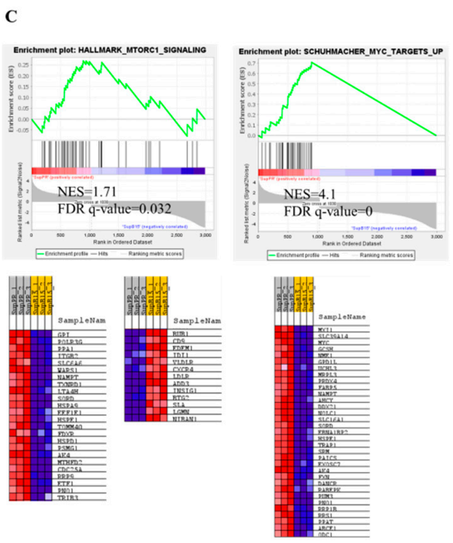


A) GO analysis network of upregulated genes in Sup-PR cells with a focus on the nodes from the cluster with most significant enrichment – metabolic processes. B) GO analysis network and annotation of downregulated genes in Sup-PR cells and a list with the top enrichment terms. C) GSEA of up- and downregulated genes in Sup-PR compared to Sup-B15 cells showing enrichment for mTORC1 and MYC signaling (NES-normalised enrichment score, FDR-false discovery rate).

Figure S2

**A** MSEA of upregulated metabolites

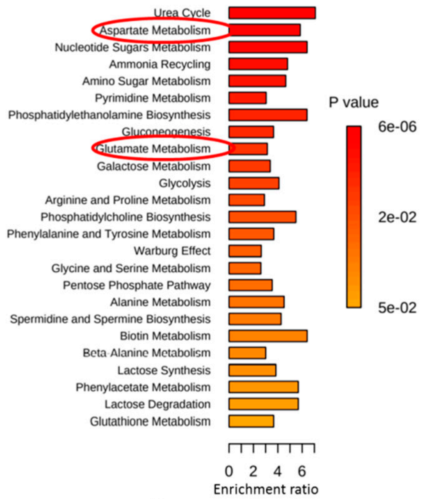


**B** Pathway analysis of upregulated metabolites

| Pathway Name | Match Status | P | FDR | Impact |
|---|---|---|---|---|
| Aminoacyl-tRNA biosynthesis | 12/48 | $7.56x10^{-10}$ | $6.351x10^{-8}$ | 0.0 |
| Arginine biosynthesis | 7/14 | $1.600x10^{-8}$ | $6.720x10^{-7}$ | 0.3655 |
| Pyrimidine metabolism | 6/39 | $4.020x10^{-4}$ | 0.0113 | 0.1349 |
| Alanine. aspartate and glutamate metabolism | 5/28 | $6.330x10^{-4}$ | 0.0133 | 0.3854 |
| Phenylalanine. tyrosine and tryptophan biosynthesis | 2/4 | 0.00396 | 0.0666 | 1.0 |
| D-Glutamine and D-glutamate metabolism | 2/6 | 0.00958 | 0.1341 | 0.0 |
| Pantothenate and CoA biosynthesis | 3/19 | 0.01241 | 0.1452 | 0.0214 |
| Amino sugar and nucleotide sugar metabolism | 4/37 | 0.01480 | 0.1452 | 0.0433 |
| beta-Alanine metabolism | 3/21 | 0.01641 | 0.1452 | 0.3993 |
| Valine. leucine and isoleucine biosynthesis | 2/8 | 0.01729 | 0.1452 | 0.0 |
| Phenylalanine metabolism | 2/10 | 0.02686 | 0.2051 | 0.3571 |

Downregulated

**C** MSEA of Downregulated metabolites

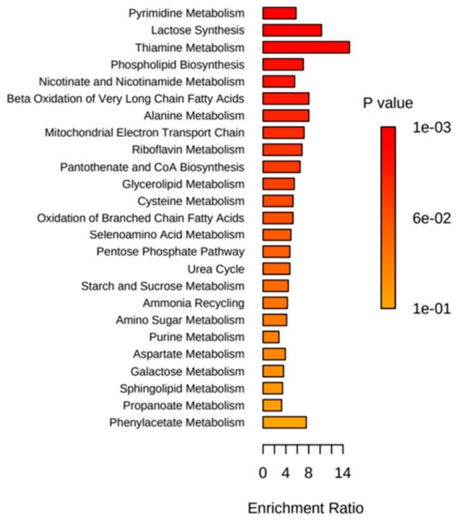


**D** Pathway analysis of downregulated metabolites

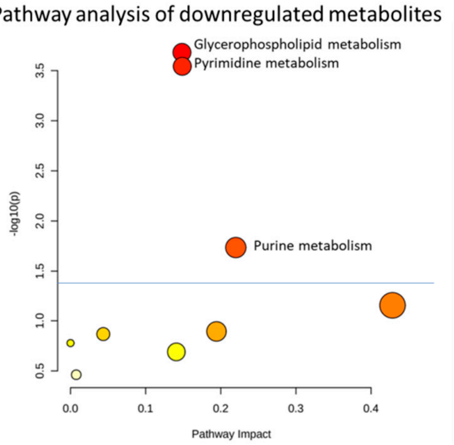


| Pathway Name | Match Status | P | FDR | Impact |
|---|---|---|---|---|
| Glycerophospholipid metabolism | 4/36 | $2.08x10^{-04}$ | $1.20x10^{-02}$ | 0.15 |
| Pyrimidine metabolism | 4/39 | $2.86x10^{-04}$ | $1.20x10^{-02}$ | 0.15 |
| Purine metabolism | 6/39 | $1.84x10^{-02}$ | $5.15x10^{-01}$ | 0.13486 |

**A)** MSEA and **B)** Pathway analysis (corresponding to figure 1D) of upregulated metabolites in Sup-PR cells compared to Sup-B15 cells showing statistical power as annotated. **C)** MSEA and **D)** Pathway analysis with a list of top hits of downregulated metabolites in the prednisolone resistant Sup-PR cell line.

Figure S3

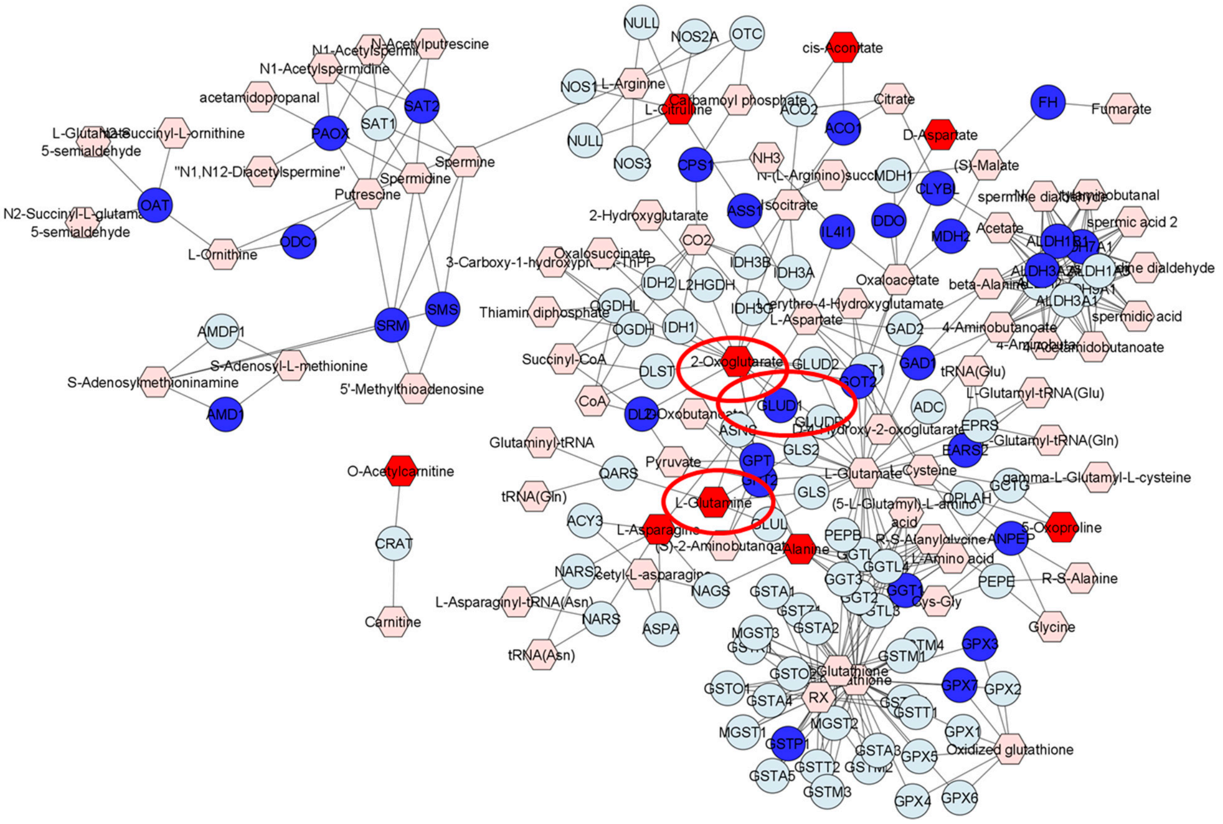


Integrated network analysis of gene expression and metabolomic data showing connections within TCA cycle with highlighted players in glutaminolysis (encircled in red – 2-oxoglutarate, *GLUD1,* and L-glutamine). Genes in our RNA-seq data and metabolites from our analysis are shown in dark blue circles and red hexagons (light blue circles and pink hexagons are for all other genes and metabolites in this pathway). Data was analysed with MetScape in Cytoscape.

Figure S4

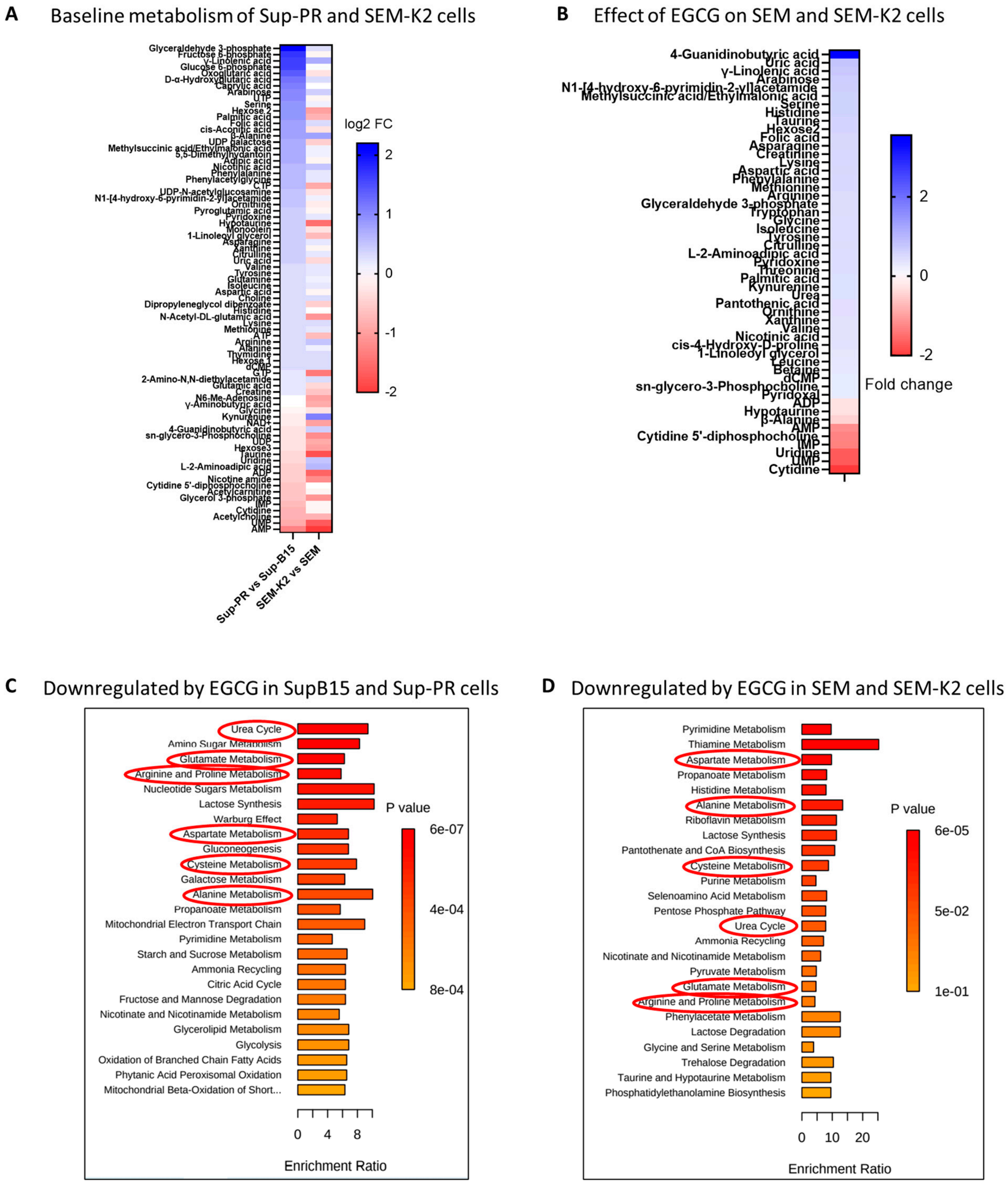


Comparison of metabolomic changes in the two sets of matched GC-sensitive and resistant cell lines (Sup-B15 and Sup-PR as well as SEM and SEM-K2). A) Dysregulation of metabolites in resistant cell lines compared to their respective prednisolone sensitive parental cell lines. B) Effect of EGCG on metabolites in SEM and SEM-K2 cells. C) and D) Analysis of the pathways of the downregulated metabolites by EGCG in the two sets of cell lines.

Figure S5

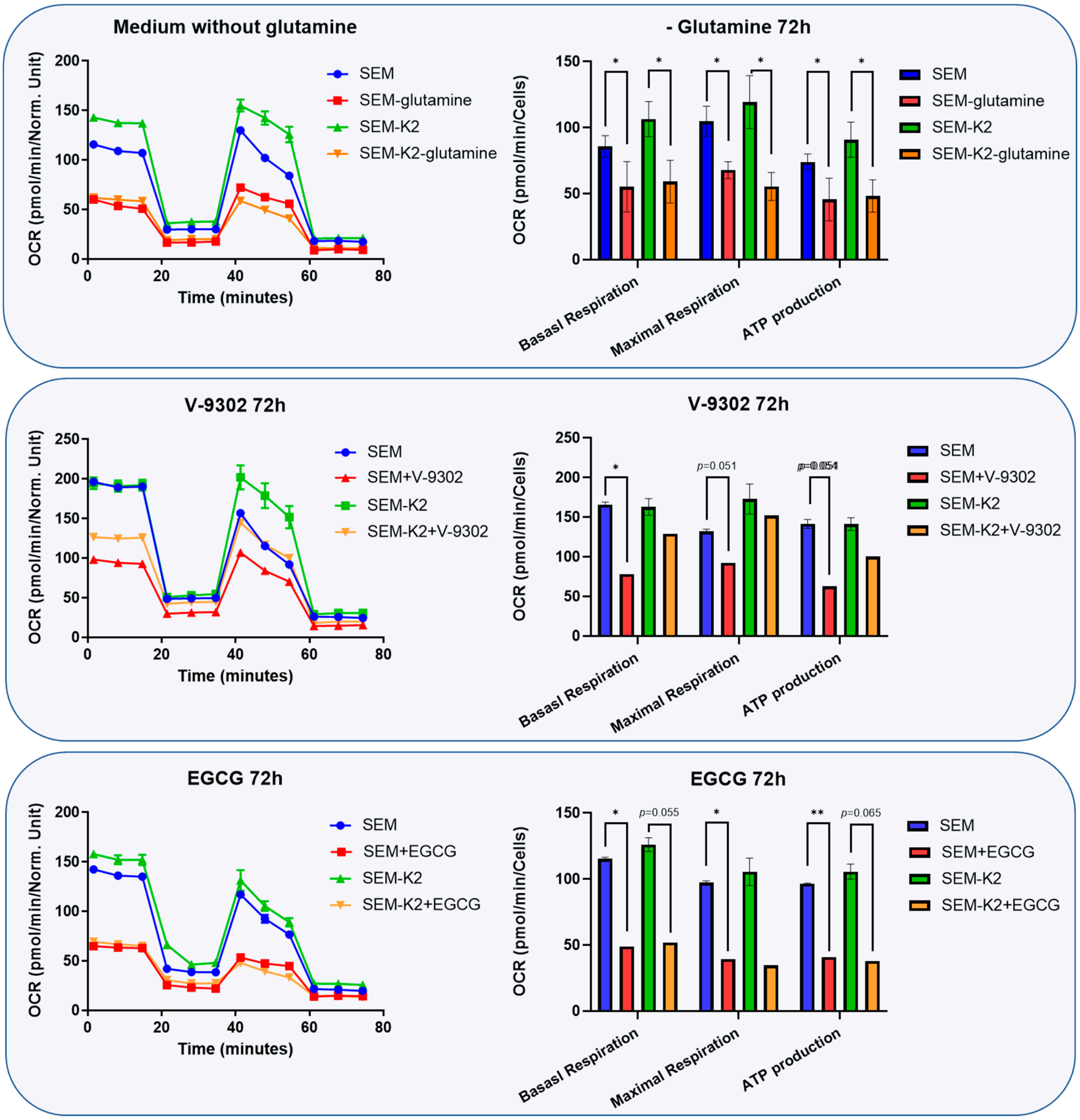


Representative Seahorse Mito Stress test graphs and analysis of SEM and SEM-K2 cells treated as annotated for 72h - grown in medium without glutamine (top panel) and treated with $IC_{50}$ values of V-9302 (middle panel) and of EGCG (bottom panel). Error bars represent mean with SD from biological duplicates. *-$p < 0.05$, **-$p < 0.005$, Student's *t*-test.

Figure S6

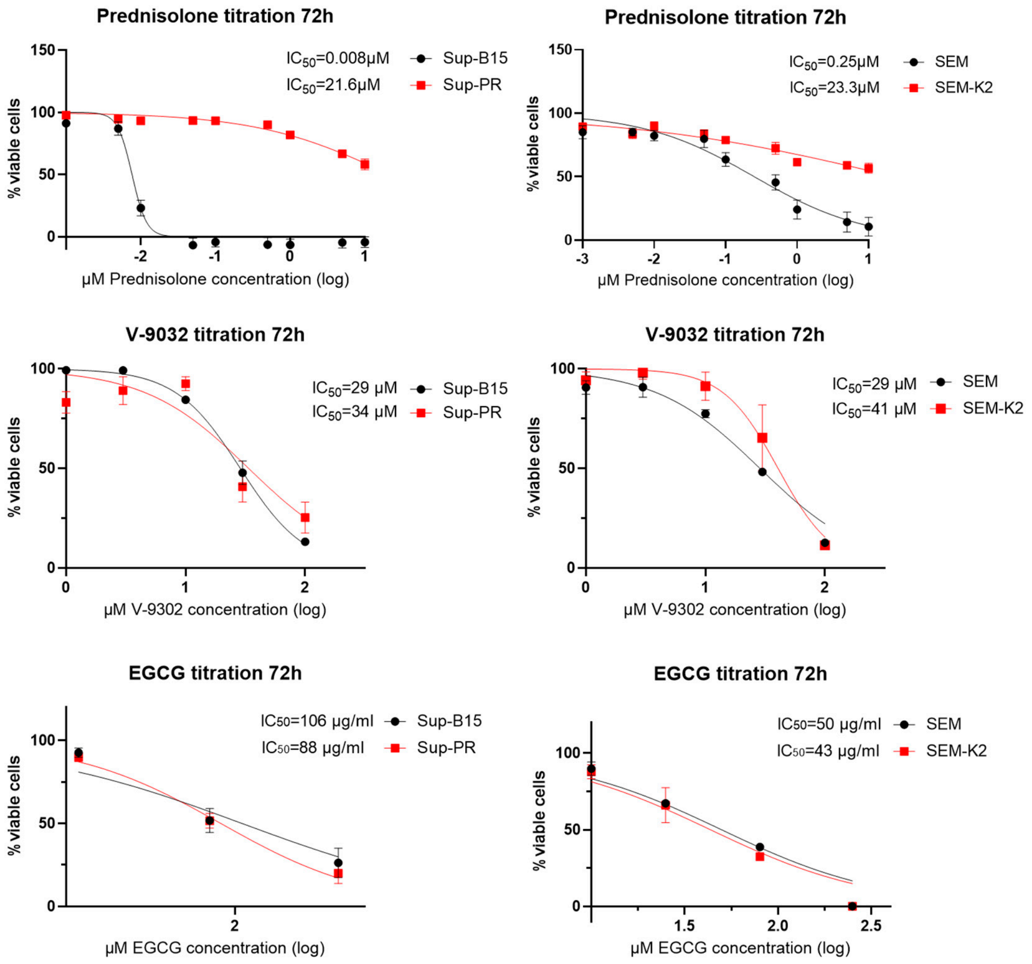


Titrations of Prednisolone (top row), V-9302 (middle row) and EGCG (bottom row) in 4 cell lines as annotated. Cells were treated for 72h and drug response was measured with MTT to calculate $IC_{50}$ value. Data present mean with SD of biological duplicates or triplicates in technical triplicates (data points show mean with standard deviation).
